# “Fox-like. One eye open, one eye closed”: child supervision among Syrian refugee mothers in Canada

**DOI:** 10.3389/fpsyg.2025.1548646

**Published:** 2025-04-02

**Authors:** Christina L. Klassen, Emilia Gonzalez, Cécile Rousseau, Jill Hanley, Garine Papazian-Zohrabian, Mónica Ruiz-Casares

**Affiliations:** ^1^School of Behavioural and Health Sciences, Australian Catholic University, Sydney, NSW, Australia; ^2^Department of Psychiatry, McGill University, Montreal, QC, Canada; ^3^School of Public Policy and Democratic Innovation, Toronto Metropolitan University, Toronto, ON, Canada; ^4^School of Social Work, McGill University, Montreal, QC, Canada; ^5^Department of Educational Psychology and Andragogy, University of Montreal, Montreal, QC, Canada; ^6^School of Child and Youth Care, Toronto Metropolitan University, Toronto, ON, Canada; ^7^Centre for Research on Children & Families, McGill University, Montreal, QC, Canada

**Keywords:** child supervision, parenting, migrant families, resettlement, Canada, Syrian refugees

## Abstract

When children live through violent conflict and forced displacement, the associated disruptions in their environment can profoundly affect their wellbeing and development, undermining stability and family cohesion essential for healthy growth. Adequate child supervision is an important component of supportive parenting but is understudied in the refugee migration context. Guided by the United Nations Convention on the Rights of the Child (CRC) (1989), which emphasizes the protection, provision, and participation of children as rights-holders, this study explored how Syrian refugee mothers resettled in Canada between late 2015 and 2017 perceived and practiced child supervision. Using a cross-sectional, qualitative design, we conducted semi-structured interviews with 20 mothers (half government-assisted refugees and half privately sponsored refugees) to examine their parenting across four migration stages: pre-conflict Syria, pre-flight conflict Syria, transit in various countries, and resettlement in Canada. Participants came from diverse religious and cultural backgrounds and spent varying times in transit (between 2 months to 5 years). Mothers’ narratives revealed how their approaches to children’s provision, protection, and participation evolved, shaped by material resources, social networks, and risks at each stage. Grounded in a critical children’s rights framework, the analysis of mothers’ daily negotiations highlights the dynamic and context-dependent nature of children’s rights, and the interconnections and tensions between provision, protection, and participation in child supervision. This study contributes to a deeper understanding of how refugee mothers navigate and uphold children’s rights throughout migration trajectories, advocating for policies and interventions that recognize these dynamic processes and the critical role of caregivers in ensuring children’s dignity and wellbeing.

## 1 Introduction

When children live through violent conflict and forced displacement, the associated disruptions in their environment can have a considerable negative impact on their wellbeing and development given the importance of stability and family cohesion for healthy child development (Harden, 2004; Negussie et al., 2019). In light of this, it is essential to understand how different components of parenting can help buffer against the loss in stability experienced by families affected by war and conflict, including refugee families. Worldwide, the number of people who have been forcibly displaced is higher now than it has ever been at approximately 117.3 million, 37.6 million of whom are refugees (UNHCR, 2024a). Supportive parenting, including adequate supervision, has been shown to be an important factor in promoting children’s wellbeing in contexts of conflict and migration (Tol et al., 2013). However, no research has yet looked specifically at changes in child (sometimes called parental) supervision across the different stages of refugee migration.

The United Nations Convention on the Rights of the Child (CRC) (United Nations, 1989) proposes a series of articles that aim to ensure the protection, provision, and participation of children globally. A children’s rights framework shifts from seeing children as ‘victims’ towards rights-holders whose dignity and integrity must be recognized and respected (Doek, 2009). The CRC states that children have the right to protection, [e.g., as refugees (Article 22), from times of war (Article 38), and from neglect (Article 19)], the right to provisions [e.g., appropriate treatment for psychological recovery and social integration (Article 39), education (Article 28) and health care (Article 24)], and the right to participation [e.g., voice an opinion that is taken seriously (Article 12), freedom of expression (Article 13), and play (Article 31)] (United Nations, 1989). While the CRC is an important starting point towards improving children’s lives, many critical children’s rights scholars argue that the international convention needs to be embedded and adapted in response to the economic, cultural, and social contextual factors and everyday lived realities of children to have meaningful and sustained influence (Reynaert and Roose, 2015). Children’s rights perspectives have previously informed decision-making in research, policy, and practice around children’s supervision, wellbeing, and development in various stages of forced migration (McAdam, 2010; Ruiz-Casares et al., 2010).

The Syrian crisis has been one of the most severe humanitarian emergencies globally over the last two decades. The civil war, which began in 2011, forced millions of Syrians to flee their homes. To date, over 60% of Syria’s pre-war population, approximately 13.7 million people, have been displaced (UNHCR, 2024b). This includes around 6.8 million Syrians who are refugees in neighboring countries (IOM, 2024) and 7.2 million internally displaced within Syria (OCHA, 2024). Yet other Syrians sought safety and better living conditions further away, often facing complex and challenging journeys to reach their destinations (Hébert et al., 2018; Belabbas et al., 2022). Since November 2015, Canada has welcomed more than 100,000 Syrian refugees (Global Affairs Canada, 2024), over 15% of them in Quebec (Government of Canada, 2024). These refugee families navigated four stages of migration: country of origin (i.e., Syria before the war); pre-flight, conflict contexts (i.e., Syria during the war); transit and forced migration, sometimes called flight or preimmigration stages (i.e., flight from Syria, time in transition countries such as Lebanon); and resettlement (i.e., Canada) (Williams, 2010; Hadfield et al., 2017). Each stage can degrade the realization of children’s rights in different ways, bringing its own stressors to families’ parenting processes and potentially having long-term effects on parents’ and children’s mental health and wellbeing (El-Khani et al., 2016; Dalgaard and Montgomery, 2017) as well as on their relationships (Betancourt et al., 2015). Grounded in a critical children’s rights framework, our study explored how Syrian refugee mothers resettled in Canada since 2015 perceived and experienced child supervision and how their ideas and practices towards promoting their children’s rights evolved across their refugee migration trajectories.

Studies have shown that children who have lived under war and conflict are at high risk for developing posttraumatic stress disorder (Baddoura and Merhi, 2015), depression (Thabet et al., 2004), anxiety (Attanayake et al., 2009), and psychosomatic problems (Özer et al., 2013). Research with Syrian refugee children and adolescents who have lived through the pre-flight/conflict and transit stages of migration has confirmed the relevance of these findings to this group (Sirin and Rogers-Sirin, 2015; Ghumman et al., 2016), meaning that many of the Syrian refugee children resettling in Quebec and Canada may face such mental health difficulties (Hadfield et al., 2017).

Family environment and parental care influence how well conflict-affected children cope with environmental stressors and psychological distress (Betancourt and Khan, 2008; Thabet et al., 2009; Panter-Brick et al., 2011). Loving, supportive, and caring parenting can promote children’s psychological wellbeing in contexts of war and violence (Qouta et al., 2008; Eltanamly et al., 2019) as well as in the often stressful context of refugee resettlement (McMichael et al., 2011). Thus, in seeking to improve refugee children’s mental health and wellbeing, interventions should focus on and address parenting practices and needs (Tol et al., 2011).

Research with Syrian refugee families in the transit/flight stage of migration has highlighted how environmental and parent-specific challenges, such as psychological distress, contribute to maladaptive parenting practices and parental concerns about their ability to parent effectively (El-Khani et al., 2016, 2017). Resettlement also brings challenges to parenting, including financial struggles, reduced social support due to distance from family, friends, and supportive relations, lack of familiarity with public services and common sociocultural practices, and parent–child role reversal (Dachyshyn, 2008; Dalgaard and Montgomery, 2017; Al Mhamied, 2023; Al Mhamied et al., 2023). Child supervision is an important component of healthy parenting; lower levels of certain forms of child supervision have been related to poor mental health outcomes for adolescents (Arat and Wong, 2016) and inadequate supervision is the most common form of child neglect in Canada (Trocmé et al., 2010). Given the large number of recently resettled Syrian refugees in Canada, there is an urgent need to investigate how each stage of the migration experience and parental psychological state have shaped parents’ attitudes and practices regarding parenting, to best support refugee children and their families as they adjust to life in Canada.

Adequate child supervision generally requires watching children closely enough to prevent serious harm, providing adequate substitute childcare when necessary, protecting children from potentially nefarious third parties, and preventing children from engaging in dangerous activities (Coohey, 2003). Child supervision can also be seen as a way of expressing care for children (Knutson et al., 2005; Duncombe et al., 2012). The concept of what is appropriate supervision and the specific ways of ensuring children are adequately supervised vary across cultures and can be influenced by migration contexts (Klassen et al., 2020). Tol et al. (2013) demonstrated the importance of child supervision and parental support through a systematic review of quantitative and qualitative studies on resilience and mental health among children and adolescents in armed conflict settings in low- and middle-income countries; this was a key moderating factor in protecting and promoting mental health.

Although statistics on unaccompanied minors in refugee migration are readily available (Bhabha and Abel, 2019), and there has been serious study on the effect of the absence of parental supervision on refugee minors’ mental health (Huemer et al., 2009; Mitra and Hodes, 2019), there is a dearth of literature specifically focusing on child supervision practices across the stages of refugee migration in situations where parents are present. Research investigating how and in what contexts child supervision is practiced and changes across the stages of migration is therefore important in order to better support refugee families at all stages of migration, including in resettlement contexts such as Canada.

## 2 Materials and methods

### 2.1 Study design

The study used a cross-sectional, qualitative research design, which allowed participants to voice their experiences in their own words, facilitating a rich account of their migration and parenting experiences (Green and Thorogood, 2013). These retrospective accounts represent a reconstruction of the past that can provide some insight into the ways participants cope with the past and/or the present, and have been employed in other studies on parenting experiences and migration experiences (Ward and Styles, 2005; Colville et al., 2009).

### 2.2 Participants and recruitment

Participants were recruited through the Canadian Institutes of Health Research-funded Refugee integration and long-term health outcomes in Canada (SyRIA.lth) project’s Montreal site’s existing database of Syrian refugee adults. SyRIA.lth is a longitudinal study of resettled Syrian refugees’ settlement strategies and social integration. The SyRIA.lth team identified participants who met the eligibility criteria for this study and who had previously agreed to be contacted for participation in qualitative studies during the course of the project. Eligible participants were then contacted by telephone by the first author and a SyRIA.lth project interviewer who also acted as a co-interviewer for this study. During recruitment, eligible participants were provided with information about the study and their rights as potential interviewees and then invited to participate.

A sample of 20 Syrian refugee mothers was recruited, with half of participants being government-assisted refugees (GAR) and half being privately sponsored refugees (PSR) (IRCC, 2020) who had resettled in Quebec since 2015 (see Table 1 for more details). GAR are selected and supported (financially and in terms of integration) by the Canadian government while PSR are identified by Canadian individuals, families, and groups that meet certain criteria, sometimes through the help of a NGO, who then take responsibility for the PSR’s living expenses in the first year and are expected to support the PSR’s social integration. GAR and PSR often have different migration trajectories prior to resettlement. Previous research has shown that PSR may experience more successful integration in Canada than GAR (Beiser, 2003). Recent studies with Syrian refugees resettled in Canada have also shown that GAR tend to be more socially isolated (Hanley et al., 2018; Hynie et al., 2019). The choice was made to select half of participants from each group in order to capture these elements of diversity within parenting experiences. Half of participants were Muslim (*n* = 10) and half Christian (*n* = 10) and came from diverse backgrounds in terms of level of religiosity and ethnic group within their respective religion (see Table 1 for more details).

**Table 1:** Study participant demographics.

	Government-assisted refugees (*n* = 10)	Privately sponsored refugees (*n* = 10)
Religious background	Muslim (*n* = 9) Christian (*n* = 1)	Muslim (*n* = 1) Christian (*n* = 9)
Ethnic background	Arab (*n* = 9) Kurdish (*n* = 1)	Arab (*n* = 7) Armenian (*n* = 1) Assyrian (*n* = 1) Syriac (*n* = 1)
City of origin in Syria	Aleppo (*n* = 3) Damascus (*n* = 1) Daraa (*n* = 1) Homs (*n* = 1) Raqqa (*n* = 1) Countryside (“Rif”)* (*n* = 3) Rif Aleppo (*n* = 1) Rif Hasakeh (*n* = 1) Rif between Raqqa and Aleppo (*n* = 1)	Aleppo (*n* = 2) Damascus (*n* = 4) Countryside (“Rif”)* (*n* = 4) Rif Aleppo (*n* = 1) Rif Damascus (*n* = 2) Rif Hasakeh (*n* = 1)
Transit countries	Lebanon (*n* = 6) Jordan (*n* = 2) Egypt (*n* = 1) Turkey (*n* = 1)	Lebanon (*n* = 10)
Time in transit country	1–12 months (*n* = 1; Turkey) 1–3 years (*n* = 4; Jordan, Lebanon) 4–5 years (*n* = 5; Egypt, Jordan, Lebanon)	<1 month (*n* = 5) 1–12 months (*n* = 3) 1–3 years (*n* = 2, both for 3 years)
Child ages	1.5 months to 37 years	
Number of children	1–7 (3 average)	

*“Rif” was the term participants used when specifying the general countryside area they lived in, if they lived outside of a major city – e.g., in the countryside outside of Aleppo was referred to as “Rif Haleb” (Aleppo’s countryside or outskirts).

To be included in the study, mothers must have had at least one child who was born between 1999 and 2011, when the Syrian civil war began (i.e., no older than eighteen at the time of the interview) (UNHCR, 2017). Mothers had between one and seven children each, and on average had 3 children; ages ranged from one and a half months at the time of interview to 37 years of age. Despite this variety, each mother had at least one child who was school-aged (i.e., between the ages of seven and eighteen). Five of the 20 families had (a) child(ren) of only one sex (female only = 3, male only = 2); only one family had an only child. Participants were from different cities in multiple regions of Syria (see Table 1 for a list) and had diverse educational and socioeconomic backgrounds, ranging from those who had not completed high school and had never worked outside the home to those who were university educated and worked in public institutions. Twelve out of 20 spent more than 6 months in a transit country; 11 out of 20 spent 2 years or more. Of the 10 GAR participants, nine spent more than 2 years in a transit country (range for GAR = 2 months to 5 years). 80% of all mothers passed through Lebanon as a transit country, and others passed through Jordan (*n* = 2), Egypt (*n* = 1), and Turkey (*n* = 1). All PSR mothers passed through Lebanon as the country of transit. Mothers left Syria between 2011 and 2015, and arrived in Canada in or after 2015. Recruitment stopped after 20 participants as this was the point when data saturation was achieved (Guest et al., 2006).

### 2.3 Materials and procedures

In 2018, individual, semi-structured interviews (Seidman, 2013) were conducted with Syrian refugee mothers to discuss their parenting and childcare experiences across the four stages of refugee migration. Interviews were conducted by the first author and the primary interviewer, who is a Syrian psychologist born and raised in Damascus and native speaker of Levantine Arabic to ensure optimal understanding of the cultural and Arabic dialect heterogeneity present within the Syrian refugee diaspora (Hassan et al., 2015). The first author (fluent in conversational Levantine Arabic) probed as necessary and took notes with special attention to participants’ facial expressions, gestures, and the surrounding environment. All interviews were audio recorded with permission, transcribed verbatim, and translated into English. Interviews took place in participants’ homes for all but two participants, one of whom was interviewed in her friend’s home, and the other in a neighborhood cafeteria of her choice.

### 2.4 Analysis

NVivo 11 (QSR International, 2015) was used to conduct thematic analysis of interview results and to facilitate both a deductive and inductive approach to data analysis (Braun and Clarke, 2006; Vaismoradi et al., 2013). Themes were developed based on interview data and analyzed according to the four stages of refugee migration (country of origin, pre-flight/conflict, flight/transit, and resettlement) described by Williams (2010); non-verbal data were incorporated in the analysis process through the use of annotations in NVivo and were considered in developmental discussions with the research team. Codes were developed manually and then entered into NVivo 11, where they were applied to interview transcripts and analyzed collectively to assist the development of themes. Multiple codes were applied to the interview transcripts, including those around parenting and supervision, which form the basis for this paper. Coding was carried out by the first author and triangulated through involvement by the third and sixth authors in reading selected transcripts to aid in code book development and to verify the representativeness of themes. The flexible approach afforded by thematic analysis (Braun and Clarke, 2006) as well as its ability to allow the researcher to analyze participants’ meaning within their own context (Joffe and Yardley, 2004) make it well-suited to this research project.

The level of analysis combined both emic and etic approaches, in that interviews were structured to discuss parenting practices more broadly and so results on child supervision were described in the participants’ own words, with the researchers determining what practices could be categorized as pertaining to supervision for the purposes of analysis. This was done by the first and sixth authors, with the sixth author bringing expertise in the area of child supervision. This approach stemmed from a desire to communicate participants’ experiences in their own voices as much as possible (Green and Thorogood, 2013). As outlined by Green and Thorogood (2013), analysis therefore began as soon as the first data were collected and was shaped by an iterative and reflective process during the research, with such reflexivity being a key element in ensuring the confirmability of qualitative research findings (Ahmed, 2024). Reflection was carried out in particular through discussion between the first author, who had prior experience working with Syrian refugee families in Lebanon, and the Syrian primary interviewer. Additionally, an advisory committee member for the research project was a Lebanese-Canadian scholar with extensive experience working with Syrian families, and she was consulted during the analysis process. In analysis, themes from interviews were contextualized by information gleaned from the first author’s observational notes during interviews (e.g., around observable signs of religiosity, mothers’ engagement with any children present, among others). Such persistent observation is essential to the conduct of credible qualitative research (Dodgson, 2019). Demographic data, such as socioeconomic status before and after migration, city and/or region of origin in Syria, language(s) spoken, and religious-cultural group, were also considered when comparing results and developing themes, given Syria’s demographic diversity, and the fact that each of these can provide insight on participant identity and background (Hassan et al., 2015).

The positionality of the interviewers was considered during analysis as well, given the first author’s identity as an unmarried, white Canadian graduate student, and the primary interviewer’s identity as a French-PhD-educated unmarried Syrian woman born and raised in Damascus, which could evoke a sense of social distance when participants were less educated or came from more rural regions of Syria. Care was taken during the interviews to find common ground with participants while maintaining a professional, ethical distance, and all participants appeared to enjoy the interview process, with many inviting the researchers to visit again.

### 2.5 Ethical considerations

There was a small risk of refugees experiencing psychological distress when discussing events surrounding migration and their new environment in Canada, and how these influence parenting. Given that sensitive topics could emerge, all necessary measures were taken to conduct interviews in a supportive environment and to connect participants to services as needed. The first author and the primary interviewer both have experience working with Syrian refugees discussing difficult experiences surrounding refugee migration and both were trained on how to sensitively ask questions and respond to distress from participants. Due to the semi-structured nature of the interviews, participants were able to choose which elements of their migration and parenting experiences they wanted to share and in how much detail. Ethnic and religious backgrounds, presented in Table 1, were not directly queried unless it became relevant within the interview, it appeared clear that the participant would be comfortable discussing this, and the participant freely offered this information. Ethnoreligious identity is often linked to political views (by presumption or in fact) and is considered sensitive information by some Syrians, particularly in the context of the civil conflict. The first author, based on her lived experience of work and relationship with Syrian colleagues and friends in the Middle East, and in consultation with her Syrian co-interviewer and advisory committee, deemed it best practice to elicit ethnoreligious identity information in this way. As such, participants’ ethnic identity was presumed as Syrian Arab unless it was made clear that they belonged to another ethnic group. This is also why specifics of Muslim and Christian identity are not provided (unless linked to ethnicity; e.g., as in the case of Syriac Christians).

No participants exhibited acute distress, yet participants were offered a list of mental health resources in the Montreal area. Ethics approval was granted by the Institutional Review Board (IRB) of the McGill University Faculty of Medicine and Health Sciences. Pseudonyms are used throughout the manuscript to preserve participant confidentiality.

## 3 Results

Mothers’ descriptions of their child supervision experiences are grouped according to the stage of migration, from Syria prior to conflict, through experiences in Syria during conflict and time in transit countries, to mothers’ experiences in the stage of resettlement in Canada’s greater Montreal area.

### 3.1 Syria prior to conflict

In Syria prior to conflict, family relationships were described almost uniformly as very close, both within the immediate and extended families. Families often engaged in leisure activities together, as one participant, Reem, said: “In Syria, (…) wherever we go there are [children] with us” and mothers were overall in charge of the home and children:

Before the war, like we were living, like happy, comfortable, my kids were young. You know, the mom, when she has control over the kids and the house, all of it (Ibtissam)

Friday and Saturday we would go to restaurants, we would take [the children] out to play, we would go to pools, we would go, (…) like you saw our social relationships, like uncles, aunts, their grandparents, and so on (…) All of them in Aleppo I mean, and these were our habits (…) like my parents were right next to me, like I used to go about 20 times a day to see my parents (Youmna)

The fact that mothers were often at home was perceived as facilitating child supervision, as was the fact that most families had parents and other relatives living close by who could help watch children when mothers held employment or otherwise could not be at home:

Some days, (…) I could not take [the children] with me. I would leave them at home. I gave my mother the key. (…) She would come to the house. (…) She knows they’re awake now, she would come to the house and see them (Fatima)

Before the war, although some mothers shared that children would diligently inform parents of their whereabouts, families generally felt safe to let their school-aged children play with less parental supervision outdoors, in extracurricular activities, and with neighbors, relatives, and friends. Familiarity with the community, environment, and people (friends, relatives, and neighbors) was described as playing a significant role in this comfort.

[The children] would go downstairs to play (…). They would ride their bikes, yes! Like we had no problems, my son was registered in a horse-riding club, he would come and go, the club's bus would take him and bring him. The other one was registered in basketball, he would come and go, my daughter, I registered her in gymnastics, like I would take her and bring her, like there was no problem at all (Youmna)

Here, there's a difference, like in our community in Syria we used to know everyone, I used to know the neighbors, if you told me I'm going to my friend, (…) I don't have a problem because I know her, and I know her parents (Farah)

### 3.2 Syria during conflict

As conflict began to affect participants’ cities and regions, this comfort disappeared. Whereas, before the war, “if [your son is] out too late, at night, you get worries,” after the war began, “you would get scared” (Ibtissam). A common concern among mothers with teenage sons was that they would be apprehended and forced to join the military, as happened to one mother who had left for Lebanon with her family when her son went back to Syria to celebrate Eid in Damascus.

The challenges brought by the conflict forced some families to choose between their priorities of family unity and providing for their children’s future through education, as well as between their attachment to home and community and safety for their families. During the conflict, some mothers shared how social support from neighbors changed from assistance with informal child supervision to updates on which areas of their cities were safe and free from missiles, shells, and other dangers.

Throughout this period, mothers shared how their social habits were completely disrupted: even visiting family down the street became dangerous, activities with extended family all but disappeared, children were no longer permitted to move anywhere alone, and mothers often shared the domestic space with their husbands full time. The level of threat participants experienced varied in severity and influenced the extent of disruption in these social habits. Only a few mothers described situations of immediate threat (e.g., daily bombings or attacks, warzone context), while more mothers described simply living under the threat of violence (e.g., the possibility of bombings or attacks, the potential for the area to become a warzone). Nonetheless, disruption in social habits was common to all participants. This shift in proximity and increase in shared space for many families was described both positively, as it facilitated closer supervision, and negatively. In some cases, mothers described the family as coming closer together, however, they also got upset with one another more easily. Tensions arose between children, father, and mother, for instance, as Youmna shared: “Like me, I was emotionally under pressure, and he was the same, and the kids (...) So it was always, you feel like, everyone was at each other’s throats for the smallest things.”

### 3.3 Transit countries

Families’ transit experiences were varied, with most PSR participants spending less time in transit countries and GAR spending up to 5 years in transit countries. Some participants were also internally displaced for protracted periods of time. Supervision experiences in transit varied across many factors, such as length of time in transit, socioeconomic status, neighborhood or region in the transit country, and transit country attitudes towards Syrian refugees. Participants who lived in transit countries for long periods of time often struggled with providing adequate supervision for their children. This was primarily due to lack of or diminished social support from extended family members. This, in turn, led to difficulties for some parents with learning certain parenting techniques, including those related to the provision of developmentally appropriate care. For example, one participant shared how, while living in Lebanon, she fed her infant daughter bread, causing her to choke. A neighbor who was present at the time told her that this was not appropriate for a child of her daughter’s age, and the realization that she could have harmed her child caused the mother distress and guilt. In this case, even though her eldest child was born in Syria, at that time the extended family had lived together, and her mother and other relatives had helped with and even taken over many caregiving and supervision responsibilities.

Security concerns were also present in the transit country. Fears for children’s safety caused some mothers to impose strict time restrictions or to not let their children, in particular their daughters, leave the home. Ibtissam shared that, “[I would] remain fear, fearful over them [children], even when they go out and go around and so on, I would remain worried, like I could not wait for them to get home.” Another participant was especially restrictive with her three teenage daughters due to safety concerns but sent her 15-year-old son to live in a more remote area in Lebanon’s mountains to do construction work with his uncle’s sons because he was always being beaten in the area they lived in in Beirut’s outskirts:

P: I was scared for [my girls], we locked them in the room, the older ones. I don’t let [others] see them. Locked [there] for safety's sake, because there is kidnapping in Lebanon. (…) I told him, my son, go [to the mountains], live there, the important thing is that I don’t stay scared for you.

I: He is the one who was beaten most of all?

P: Because he’s a boy, and the boy wants to go out, he can't stay and sit at home, with boys, it’s hard. Now girls, I told them, I locked [imprisoned] them in the house, but the boy, I can’t lock him up (Bouchra)

Family relationships in transit countries were impacted by tension associated with cramped physical space - for example, some families, like Bouchra’s, lived in one-room houses with multiple children and both parents. Opportunities to socialize were also described as severely limited, and one participant shared the feeling that her son’s shyness with other children in Canada was because he had had no opportunities to socialize with children his own age while living in transit in Lebanon:

When I left Syria, David was 2 years and half. He did not understand the social relationship with his age – other children. During the entire time I was in Lebanon, David was spending his time in that room. (…) He didn’t play with other kids. (…) I used to take him out, but he didn’t see kids on the street to play with. I also didn’t know anybody who had kids his age to play with. (…) When I came to Canada, I stayed 1 year or maybe 8 months staying here alone (…) Now, my son, David, has a problem with kids (Amira)

In the face of fears for their children’s safety while in insecure transit contexts, mothers were innovative in finding ways to preserve a sense of maternal authority and protection for their children, while providing them with spaces to participate in social and educational activities. When education centers were opened for Syrian refugees in Egypt, Fatima combined her need for work with her uncertainty about the schools’ distance from home to come up with a way to continue supervising her children:

Most people started complaining that (…) [the education centers were] too far away, like you don't trust to send [your children] that far. (…) so, I went and I worked and applied for a school, I worked in it as a [bus] supervisor (…) so I would bring the kids and deliver them, and I would stay sitting in the school (Fatima)

### 3.4 Resettlement in Canada

Mothers identified three common barriers to support in child supervision in Canada: Lack of trust due to not knowing friends and neighbors well, being unfamiliar with the Canadian environment, and lack of time on their and others’ parts. Mothers were hesitant about letting unknown outside influences into their families. This meant that families who had no extended family living nearby suffered from this absence, while those who had supportive, present family nearby described appreciating this support. As Rima shared, “You cannot, like, leave the children to anyone. With the family, you could – (…) They removed a big load. But here, you cannot trust for anyone to take the child.”

In addition to feeling that those outside the family or who were not well known could not be trusted to care for their children, many mothers shared about the change in pace of life that they experienced in Canada and how this impacted both their ability to parent and others’ willingness to help:

I don't know anyone I can depend on. (...) Because if I needed someone and I asked something from them, they might ponder it before they agree, they wouldn't tell me yes immediately. Even the ones we know, they're our friends, not more (...) But not because they don't like to. (...) They too are either busy with their school, or their work (Reem)

In Canada, although many mothers described challenges with caregiving, the fact that the children were physically safe eased their worries: “Even here, there is tiredness, but here, what relaxes you is, first thing, the children are psychologically comfortable from their end. They come and go.” (Fatima).

Some mothers described how, due to working in Canada, they were unable to spend as much time with their children. In some cases where the children were perceived to have gained more independence in decision-making around social activities outside the family:

They would not ask after us, they even have more freedom, they stopped taking our thoughts on everything, like me before in Syria, they would not leave the house before telling me “I’m going out to this place.” Now, here in the afternoon, I’ll find [my son] dressed up [and] I ask him “Where are you going?” He says, “I do not know” (…) What do you mean I do not know? He’ll tell me, “With my friends, maybe to the park, maybe to the mall” (Reem).

In other cases, mothers understood this independence as rebellious and attributed it to the fact that they were not physically present with their children, as in the case of Reem’s eight-year-old daughter:

P: [I]n regards to misbehavior, (…) they've really become impolite (…) because, first of all, I'm leaving them alone, (…) they're going out with their friends, and they're coming, now [my daughter] when I go to work, you see these her friends? (…) From the morning she's with them, till I come back, you know? For example, yesterday she told me "Let me go to the pool", I told her "No". She said "No, in fact I'm going” (…) This is not the only time it happens. It has happened a few times. (…)

R: and it wasn't like this in Syria

P: No, of course not (…) because I was always sitting with them (…) And now, I'm here not working 5 days, I'm working only 3 days a week (Reem)

Mothers were eager to facilitate their children’s integration, particularly through friendships, education, and employment, but wanted to do so in a way that allowed them to be fully informed on the influences that these opportunities could have.

[My 16-year-old son] told me “I want to be employed”, I told him “go sign up” (...) I would also like for him to work for example, just for a few hours so he can gain experience in this culture (...) if he got work I would let him work, but (...) under my watch (...) like I'll know where he's coming and going, "Mama," for example "today I applied here, I applied here" not by himself, he goes out in the morning and doesn't come back until evening and I don't know where the kid is… (Youmna)

[T]he parents of the friends, we go and sit in the park, like, I reassure myself that I'm watching after my own daughter, and it's a public space. Not like at someone's house or something (…) I am sitting and speaking with them and watching my daughter (…) I'm not taking away their fun, like, I let them speak to everyone. (…) and regarding to the older one, the first time she wanted to go see her friends, I went with her. Like, I visited the parents, and I saw the house, (…) I liked the situation. Like she is a normal mom, regular, a family, there's a father, like the situation was suitable for me. Like this, her relationship with her friend continued (…) The (…) places I haven't gone to, she's not allowed to go (Farah)

Like now my other son he's a bit older now, he's 16 now, so he's registered in a gym by himself (...) But my daughter no, I take her and I bring her I mean, like she tells me I want to go to the pool here for example, in the park near us (...) I'm scared (...) Yes and I go with her, for example, or there's a camp she can go with (...) if she wasn't accounted for 100% I wouldn't send her. Yes, but they have friends, and with their cousins here, like now my two sisters are here, and my two brothers are here (Youmna)

Mothers were especially eager to carefully supervise their children when it came to helping them develop strong moral and cultural values. They were aware of the influence that children’s friendships could have, for better or for worse, and wanted to be the strongest influence in their children’s character development.

I have to stay monitoring [my children]. Fox-like. One eye open and one eye closed. Even [if] I am tired in the afternoon and have fed them and I want to relax, I would like, uh, to lay down next to them. Even if it’s on the sofa. I lay down and they’re around me. I don’t feel like I’m comfortable unless they are with me. I hear what they talk about. (…) I feel safe if they are near me. The day we arrived, all of the children in the building came here. I tell them, “Stay here. Don't go out.” (…) I see them, I listen to them. You see who your children are interacting with. What if you want to turn them away, for example, from an idea they are talking about. Here, you will intervene. You want to see the children and their upbringing and you intervene as you wish, because the children always affect each other. (…) You have to stay monitoring and listening (Khawla)

Some mothers also described the ways in which they were working to help repair the negative effects of their children’s social development being interrupted during transit country and conflict experiences by modifying supervision practices to facilitate healthy social relationships. Below, Amira continues to share about her son David’s difficulties while describing how she has tailored her supervision to promote his positive social development:

This is because he did not play with other kids in the first 5 years of his life. (…) I feel he lacks something. (…) That’s why, now, I let him play a lot. (…) I watch him from the window, for example, but I do let him play as long as possible. I don’t know if this is the right thing to do – compensating for the things he lost (Amira)

Overall, mothers tied their wellbeing to their perception of their children’s wellbeing and to the wellbeing of their relationships with their children. Mothers who had older children who were unable to be in Canada tended to be distressed about this and repeatedly came back to this in interviews, with more than one mother saying they were overall happy except for the absence of their older child(ren).

Role reversal also occurred between parents and their children, and in most of the cases where this was described, it was due to children’s more rapid language learning. As one mother, Sirvart, stated, “[In Syria], [my son] was dependent on [us,] his parents. Here, no. We are the ones depending on him. ‘Come here and explain. Come here and take us there.’”

## 4 Discussion

Although the 20 mothers we interviewed came from diverse religious, ethnic, educational, and socioeconomic backgrounds, and regions within Syria, a common thread was the value they placed on their children’s wellbeing, and the crucial role they played in advocating for their child(ren)‘s rights by promoting their wellbeing, maintaining maternal authority, and uniting the family. Mothers worked to promote family cohesion through chaotic circumstances. This is promising given the accumulating evidence that family communication, family cohesiveness, and social support can protect against the negative effects of conflict on children’s wellbeing (Figley, 1983; Thabet et al., 2009). This commonality is noteworthy given the heterogeneity of the Syrian population (Hassan et al., 2015) and our own study sample. Previous studies have drawn attention to the fact that parental monitoring or supervision of children promotes positive mental health outcomes for children (Tol et al., 2013), yet these have primarily focused on supervision as one component of broader measures of supportive parenting.

Our results highlight the way child supervision was an important focus in mothers’ efforts to promote their children’s wellbeing in that, although our semi-structured interview schedule did not center on child supervision, it emerged as an important theme and topic of concern that our participants repeatedly brought up and emphasized in unsolicited ways. Across the stages of migration, participants paired supervision practices with their descriptions of family wellbeing and maternal peace of mind (or absence thereof). As they were able, mothers adapted their caretaking practices in line with the freedoms or constraints of their contexts and migration stage in order to best suit their perception of their children’s needs and to promote their own personal and relational wellbeing, including a sense of maternal authority. Throughout migration, mothers worked to maintain family connection and unity, whether across oceans or while living in one-room apartments in unsecure transit country communities. As such, our study illustrates the key role that mothers play in both confronting and transforming the evolving constraints and resources in the context of forced migration to advocate for and improve access to the rights of their children.

A children’s rights framework challenges the narrative that children and young people are ‘issues’ to be addressed and, rather, views them as rights-holders who deserve to be met with respect, dignity and integrity. Reconceptualizing children’s rights as ‘living rights’ recognizes the realization of rights as a dynamic process that influences and is influenced by economic, social, and cultural contextual factors (Hanson and Nieuwenhuys, 2013). As this article has shown, migration is also a dynamic process where children and families are faced with challenges and respond with strategies that vary depending on the constraints and resources available to them during each stage of migration. A dynamic understanding of children’s rights is then appropriate for research with migrants and can draw our attention to nuanced changes in children’s and families’ needs during their migration trajectory. In fact, the interconnection and tension between the rights to provision, protection, and participation has been widely studied in the context of children’s rights (Driskill et al., 2010; Collins, 2017; Heimer et al., 2018; Vissing, 2023). This study contributes to a more nuanced understanding of the relationship between these elements in the context of a forced migration process, particularly given many participants’ experiences of traumatic events. Such traumatic experiences and the experience of resettlement can produce varying results in parenting approaches, ranging from those that are more autonomy supporting to those that involve greater parental control (Eltanamly et al., 2022). Such control can lead to overprotection, and, whether the result of trauma or uncertainty and lack of familiarity with a new environment, this can hamper children’s active participation in public spaces, while more autonomy supporting responses tend to foster such participation. Our results indicated such variable responses to stressors throughout the migration stages, emphasizing the need for nuance when exploring families’ migration experiences. Our following discussion describes how mothers’ daily negotiations between provision, protection, and participation are shaped by the material and relational resources available in each stage of migration, as well as the (perceived and real) risk of harm.

### 4.1 Children’s rights prior to conflict

The evolution of child supervision and family relationships across the four stages of refugee migration for our sample could broadly be described as follows: Prior to conflict, mothers relied primarily on extended family members for support in supervising and caring for their children and were most often present themselves to supervise; relationships within the immediate and extended family were overall close, with mothers emphasizing the importance of family unity and time spent together, including in leisure activities. There was less concern about children’s safety in coming and going from the home compared to future stages of migration. These social and environmental conditions allowed mothers to feel more at ease about letting their children participate in social activities in more autonomous ways. Other work discussing Syrian family relationships has underscored the important role that the extended family (“ahl,” or kin) has in Syrian culture, with relatives expected to support one another with childrearing and other responsibilities (Haboush, 2005; Al Mhamied, 2023; Al Mhamied et al., 2023).

### 4.2 Children’s rights during conflict

During conflict, daily routine, security, social support, and relationships were all disrupted, patterning with other summaries of the chaos that conflict can wreak on parenting contexts (Murphy et al., 2017). Similarly to other studies showing how economic suffering and overcrowded living could lead to elevated expressions of emotions such as anger (Eggerman and Panter-Brick, 2010), our participants also described how living in cramped quarters due to security concerns or destruction of property led to family tensions and heightened supervision and protection at a level that was stressful over time. Protecting older boys from potential recruitment into war and girls from threats of dangers outside the home also limited their mobility and autonomy. Interestingly, a few participants also shared how these experiences brought the family closer together, where children were more likely to interact with close family members than with the broader community as they did before the conflict. The fact that participants experienced conflict to varying degrees, as described in the results, is important to note, as other research reviewing multiple studies on parenting in war has found that immediate threat situations are more likely to create factors leading to limited parental warmth and support, while the context of living under threat, despite creating high stress, did not leave parents wholly unable to support their children emotionally and materially. In these contexts, parents often demonstrated an increase in warmth and even overprotection, which patterns with our results (Eltanamly et al., 2019). Evidently, children’s participation and social mobility was particularly restricted during this stage in the migration trajectory. Despite family tensions, mothers played an important and continuous role in promoting family cohesion, as a means to ensure children were protected and provided for by close family members.

### 4.3 Children’s rights in transit

Mothers’ descriptions of experiences in transit echoed the challenging contexts described in other studies of Syrian refugees living in transit countries such as Lebanon (Sim et al., 2018) and results from studies bringing together research on a variety of refugee background and host country contexts (Miles et al., 2019). During both conflict and transit migration stages, supervision appears to become one of the mechanisms by which mothers feel they are able to ensure children’s safety to some degree in an insecure, uncontrollable environment. It is also one of the ways mothers in our study negotiated spaces for children to participate, whether by removing them from harmful contexts (for example, by sending them to more remote areas) or by integrating themselves into these spaces (for example, by assuming a role in the schools). The extent to which mothers are able to provide some level of stability, security, and family cohesion through supervision has important implications for children’s development, given the importance of family stability and cohesion for healthy child development (Harden, 2004; Negussie et al., 2019). Some mothers’ descriptions of their struggle to provide safe contexts for children’s participation in play and socialization in conflict and transit (e.g., Amira), primarily due to unsafe (and unfamiliar) environments and/or lack of social network, and their efforts to facilitate this securely through supervision in resettlement (e.g., Farah), similarly has implications for children’s development, as opportunities to play help promote children’s healthy development (Milteer et al., 2012; MacMillan et al., 2015). Protection plays a crucial role in shaping the resilience of young war victims by ensuring their physical safety (Shenoda et al., 2018), access to trauma-informed psychological support (Bürgin et al., 2022; Vostanis, 2024), and a stable environment (Slone and Peer, 2021). Even in the transit stage of migration, it has been demonstrated that parenting support is sought after by some Syrian refugees in camp and humanitarian settings (El-Khani et al., 2018).

The gender-related differences in negotiating between participation and protection were also noteworthy, such as the idea that boys need freedom – i.e., they cannot be kept inside – even when it is dangerous outside, whereas girls can be kept inside for protection when there is the threat of danger. This seems to pattern with results from work on children’s play in refugee migration, which showed that girls demonstrated more limited outdoor play pre-migration to Australia than boys (MacMillan et al., 2015). Although our participants did not live for protracted periods in refugee camps, the uncertainty and lack of control over their surroundings was similar to the experiences described by Syrian refugee participants in camp contexts in Turkey and Syria (El-Khani et al., 2016). These and other environmental challenges that limit children’s engagement in activities that promote their participation and wellbeing have been shown to precipitate caregiving changes such as decreased child supervision in other studies with Syrian families in transit in Lebanon (Miles et al., 2019). Future research could look more closely at the ways in which social norms and behaviors around gender roles, of both caregivers and children, influence the way children’s participation is promoted and enacted during all stages of migration.

Other studies with Syrian refugee mothers in Lebanon have demonstrated the importance that social support, specifically emotional support, can have in promoting mothers’ psychological resilience (Sim et al., 2019). Based on the distress described by many of our participants at feeling socially isolated and unsupported in Lebanon, we agree with Sim et al.’s (2019) recommendation to investigate interventions that seek to increase mothers’ access to social support in transit contexts. The few participants who described having an easier time in lengthy transit contexts were those who were also observed to most likely be of higher SES background (e.g., one participant, Myrna, whose daughter moved from a private French school in Syria to a private French school while in Lebanon). It is important to note that all our participants who lived in Lebanon for more than 2 years, with the exception of Myrna, described experiencing high levels of stress, safety concerns, and difficulty adequately supervising their children while there. Our participants were selected to come to Canada in 2015–2016 and restrictions on and stressors for Syrian refugees living in Lebanon have only increased since then due to multiple complex socio-political factors and changing laws (see Akesson and Coupland, 2018; Kerbage et al., 2019). It is thus reasonable to expect that some of the challenges of parenting in Lebanon have increased, emphasizing the need for continued attention to the challenges of Syrian refugees living in countries where permanent resettlement is unfeasible and supporting the call for increasing the number of refugees to be taken in in resettlement countries such as Australia, Canada, New Zealand, the U.K., and the U.S.A.

### 4.4 Children’s rights in resettlement

In resettlement, although some mothers were overwhelmed by the dramatic cultural shift to Canada and remained hypervigilant, it appeared that the change was more manageable and appealing over time. Some contributing factors included the opportunities that living in Canada offered their children in the long term and the overall physical security participants described. In this setting, mothers were able to shift their focus more to facilitating friendships and social and educational advancement for their children, while maintaining a comfortable level of supervision and involvement in these processes. The importance of supervision for mothers was noteworthy. Multiple mothers provided examples of how protecting and providing for their children was directly related to their sense of maternal authority, and their ability to promote family unity and other cultural values. Mothers’ reluctance to rely on non-kin carers for supervision echoes work on Arab families stating how, although highly valued, non-family relationships are never as important as those within the family (Abudabbeh, 2005; Al Mhamied, 2023) and family is seen as the first source of support (Hassan et al., 2015). It also echoes broader research on parenting stress and non-parental care, which showed that non-parental kin care caused less parenting stress for parents with children under 5 years old than non-parental, non-kin care (Craig and Churchill, 2018). Some mothers had to take up employment in Canada to help provide financially for their families. In turn, this decreased ability to supervise their children was perceived as negatively influencing the parent–child relationship while contributing to children’s poor behavior, as echoed by other work with Syrian refugee families in Lebanon (Sim et al., 2018).

An alternative perspective found by research with immigrant families in Canada has shown the complex and dynamic ways in which child agency is negotiated between parents and children in the host society, advocating for the need to consider parent–child relationships, material and social resources available, and the perceptions of safety in the host society (Gonzalez and Ruiz-Casares, 2022a). As seen in this study, immigrant children who assume the role of translators for parents is a common finding in research with immigrant families, and another example of how young people’s participation is interconnected to their access to services and protection (Lucas, 2015; Gustafsson et al., 2019). Role reversal and perceived loss of maternal authority can cause stress for caregivers, particularly in a culture where parental authority and family hierarchy are considered a meaningful part of parental identity and role (Haboush, 2005). Given this, it is important to consider how opportunities for supervision can promote a healthy authority in the midst of the parent–child role reversals that often accompany refugee migration, while also offering opportunities for children to participate autonomously in social activities and decision-making. Additionally, participating in community-based activities where children’s perspectives and interests are considered in decision-making has contributed to the wellbeing and sense of belonging of children who experienced forced displacement (Gonzalez and Ruiz-Casares, 2022b). Mental health professionals and social service providers working with families at any stage of migration can look at ways in which mothers can be supported to restore or rebuild healthy relationships with children, where maternal authority and child agency can be negotiated.

The way that mothers emphasized the fact that they were happy in Canada, except for the absence of key family members, further highlights participants’ desire for togetherness, family unity, and ability to positively influence the direction of their children’s lives and development, a finding that is in line with work with Syrian refugee families living in Turkey (Arenliu et al., 2020). This unity could be seen in some senses as an extension of supervision and a rounded out interpretation of what the emotionally supportive component of child supervision can look like. It also seems that, by supervising their children, mothers were able to decrease some of their own anxiety stemming from their forced migration experiences.

## 5 Study limitations

Some limitations of our study need to be considered. Our sample was relatively small and restricted to a handful of neighborhoods in the neighboring municipalities of Montreal and Laval, Canada, and no children, fathers or other family members were interviewed. Nonetheless, given the roles mothers typically fulfill as primary caregivers for children, and the diversity of backgrounds and balance of migration trajectories represented by our 20 participants, results still contribute toward a deeper understanding of the Syrian refugee migration experience. Small sample size also prevents us from comparing experiences based on the country of transit except for Lebanon, where over three-fourths of participants spent time prior to resettling in Canada. Furthermore, the cross-sectional approach makes it possible that participants may have transformed, idealized, or avoided some elements of the past when sharing their experiences. However, parents’ subjective perceptions and accounts of their parenting experiences are nonetheless an important data source. Since only mothers were interviewed, the information they provided could not be triangulated, except by observation of the home environment and brief interactions with other family members during the visit. Future research should examine children’s and adolescents’ perceptions of supervision and its relative importance in their parents’ support of their wellbeing in the refugee context (Eruyar et al., 2020), as well as parents’ explicit descriptions of the value of this component of parenting in maintaining their and their families’ wellbeing and cohesion. Further research with Syrian refugee fathers on their views on child supervision would also be valuable given their underrepresentation in the literature on refugee parenting (Bond, 2019), with only a single study to date examining the fathering experiences of Syrian refugees to Canada (Al Mhamied, 2023; Al Mhamied et al., 2023).

Despite these limitations, this study highlights the crucial role of child supervision in the context of parenting in migration. A key takeaway from our results is the readiness with which mothers brought up child supervisory practices when asked questions about parenting and changes in parenting across the stages of migration. The ability to supervise was linked by mothers to other factors shown to influence child wellbeing in conflict and buffer against trauma and stress, such as supportive family relationships. Syrian mothers’ efforts to provide a supportive environment and to promote their children’s rights and wellbeing through the provision of adequate supervision throughout the challenging refugee migration experience is a remarkable example of resilience in action. Strengths-based and children’s rights approaches interventions for refugee families that acknowledge and find ways to facilitate this supervision are likely to be most supportive in promoting both children’s and mothers’ wellbeing.

## 6 Conclusion

Through the different stages of forced migration, we have described some of the interconnections and tensions between provision, protection, and participation of children’s rights, and the crucial role mothers play in advocating for and upholding these rights. Our study shows how most mothers are able to adapt their parenting practices in response to contexts of elevated risk and fear, and then, while remaining a bit hypervigilant in the host society, move back to a more open model favoring socialization. We also cast light on how evolving caregiving practices and norms, such as overprotection, gender roles, and family dynamics, affect mothers’ negotiation of protection and participation at different stages of the migration trajectory. These results invite us to trust refugee parents’ judgements and skills, and to read what may be considered inappropriate protectiveness or lack of supervision as possibly related to the past or present family predicament. In general, it is important that interventions for refugee children and families be informed by an awareness of the diverse benefits that providing adequate supervision can have for overall family wellbeing, such as by supporting healthy family relationships (e.g., by promoting family unity) and maternal wellbeing (e.g., by helping mothers regain and/or retain a sense of agency in uncontrollable circumstances), as well as by promoting child mental health and wellbeing (e.g., by providing for children’s safety and creating a sense of stability in chaos). In this process, it is also important for both professionals and parents to be aware of the negative impact that overprotective parenting can have on children’s mental health, as shown by work with Syrian refugee children in Turkey (Eruyar et al., 2020).

To support mothers and children in transit and resettlement countries, it is essential to adopt strengths-based and children’s rights approaches. These interventions should acknowledge and facilitate maternal supervision while promoting both children’s and mothers’ wellbeing. Balancing children’s rights to provision, protection, and participation is crucial. Mothers, often overprotective due to traumatic experiences, need support to understand and respect their children’s participation rights, especially during adolescence. This balance can be achieved by educating mothers on the importance of children’s autonomy and involving children in decision-making processes. Specific children’s rights, such as the right to be heard (Article 12 of the UNCRC), the right to protection from harm (Article 19), and the right to education (Article 28), are vital in designing this framework. These rights ensure that children’s voices are considered, their safety is prioritized, and their developmental needs are met, fostering a supportive environment for both mothers and children (Alshammari et al., 2025; United Nations, 1989). Furthermore, the implementation of children’s rights needs to consider children’s everyday lived realities, as was shared from the perspectives of the mothers in this study. Future research should consider the perspectives of children and young people themselves. Additionally, further research exploring the sense of time moving more quickly in Canada than in Syria, as well as children’s tendency to adapt more easily and quickly in the host society than their parents, and how both of these influence parental self-efficacy, social support, and ability to provide adequate supervision, would also be of interest.

## Data Availability

The datasets presented in this article are not readily available due to ethical restrictions. Requests to access the datasets should be directed to Christina L. Klassen, christina.klassen@myacu.edu.au.
